# Association of sleep disturbance with risk of cardiovascular disease and all-cause mortality in patients with new-onset type 2 diabetes: data from the Korean NHIS-HEALS

**DOI:** 10.1186/s12933-020-01032-5

**Published:** 2020-05-13

**Authors:** Young Choi, Jae Woo Choi

**Affiliations:** 1 Department of Health Care Management, Catholic University of Pusan, 57 Oryundae-ro, Geumjeong-gu, Busan, South Korea; 2 College of Pharmacy, Yonsei Institute of Pharmaceutical Sciences, Yonsei University, 162-1 Songdo-Dong, Yeonsu-Gu, Incheon, South Korea

**Keywords:** Mortality, Cardiovascular disease, Coronary heart disease, Sleep disturbance, Stroke, Type 2 diabetes

## Abstract

**Background:**

Sleep disturbance has been significantly associated with the incidence of cardiovascular disease (CVD) in the general population. However, despite the common prevalence of sleep disturbance in patients with type 2 diabetes, its relationship with the risk of CVD remains unclear. Here, we have examined the association of sleep disturbance with the incidence of all CVD and all-cause mortality in patients with newly-diagnosed type 2 diabetes.

**Methods:**

We used the Korean National Health Insurance Service–Health Screening Cohort data and included 36,058 patients with new-onset type 2 diabetes aged ≥ 40 years between 2004 and 2007, along with follow-up examinations to 2013. We used the ICD-10 code to measure sleep disturbance as a primary diagnosis and the multivariable Cox proportional hazards regression models to estimate the adjusted hazard ratio (AHR) and 95% confidence interval (CI) of all CVD, coronary heart disease (CHD), stroke, and all-cause mortality.

**Results:**

We identified 6897 cases of all CVD (CHD, n = 4138; stroke, n = 2759) and 2890 events of all-cause mortality during a mean follow-up period of 7.0 years. Sleep disturbance was associated with an increased risk of All CVD (AHR, 1.24; 95% CI, 1.06–1.46), CHD events (AHR, 1.24; 95% CI, 1.00–1.53), and all-cause mortality (AHR, 1.47; 95% CI, 1.15–1.87) in patients with new-onset type 2 diabetes. Furthermore, women (AHR, 1.33; 95% CI, 1.06–1.67) and middle-aged adults (AHR, 1.29; 95% CI, 1.02–1.64) with sleep disturbance had a significantly increased risk of CVD than those without; contrarily, men (AHR, 1.45; 95% CI, 1.09–1.95) and older adults (AHR, 1.51; 95% CI, 1.15–1.99) with sleep disturbance were associated with a significantly increased risk of all-cause mortality than those without.

**Conclusions:**

Our findings suggest that sleep disturbance is significantly associated with an increased risk of CVD and all-cause mortality in patients with new-onset type 2 diabetes.

## Background

There were approximately 463 million patients with diabetes globally in the year 2019, which may increase to 700 million by 2045 [1]. The global economic burden of treating diabetes was estimated to be 1.31 trillion USD/y, considering both the direct costs and production losses due to disease morbidity or premature mortality [2]. This outcome consequently indicated that diabetes has become a silent epidemic on a global scale and poses a serious health concern [3]. Despite the advantages of early diagnosis and improved treatment modalities, the risk of cardiovascular disease (CVD) in patients with type 2 diabetes remains remarkably higher than in those without diabetes [4].

Globally, CVD is considered to be the leading cause of premature mortality and accounts for more than 330 million years of life lost per year [5]. In Korea, 62,947 deaths were attributed to CVD, which accounts for more than 21% of the total deaths in the year 2018 [6]. Considering the fact that type 2 diabetes increases the risk of CVD by more than two-fold [7, 8], along with the increasing number of patients with type 2 diabetes and the substantial economic burden of diabetes, it is imperative to investigate the modifiable factors associated with the risk of CVD in patients with type 2 diabetes [3].

Sleep is indispensable to human health and an individual typically spends approximately one-third of his/her life sleeping. It is considered to be a lifestyle behavior that can significantly influence the incidence of CVD and death [9]. A recent global prospective cohort study reported a J-shaped association between the measured duration of total daily sleep and mortality and major cardiovascular events [10]. A number of meta-analyses also demonstrated that sleep problems such as excessive (short or long) sleep durations or poor sleep quality may increase the overall risk of CVD and all-cause mortality [11–15] and the results suggest that sleep management may be essential in preventing CVD.

Sleep disturbance is common in patients with type 2 diabetes [16] and a previous study reported the high prevalence of sleep dysfunction in patients with type 2 diabetes, and demonstrated that a high proportion (69%) of type 2 diabetics had a Pittsburgh sleep quality index score ≥ 5, suggesting reduced and disturbed sleep [17]. However, there is limited evidence on the association between sleep disturbance and the risk of incident CVD in patients with type 2 diabetes. Although a cross-sectional study suggested that poor sleep quality is considered as a potential risk factor for CVD in 332 patients with type 2 diabetes [18], the findings only indicated a bidirectional association between sleep problems and CVD in patients with type 2 diabetes.

Therefore, we have examined the association of sleep disturbance with the incidence of all CVD, coronary heart disease (CHD), stroke, and all-cause mortality in patients with newly diagnosed type 2 diabetes using the nationwide population-based cohort data. Additionally, we have identified the association between sleep disturbance and risk of all CVD and all-cause mortality stratified by sex and age.

## Methods

### Data and study sample

We used the Korean National Health Insurance Service–Health Screening Cohort (NHIS-HEALS) data [19]. The Korean NHIS operates an obligatory health insurance program and that covers approximately 97% of the entire Korean population. All subscribers aged between 40 and 79 years are required to undergo a standardized, biannual health examination. The sample of NHIS-HEALS was extracted by a simple random sampling method to develop a representative sample that included 10% (514,866 individuals) of all health screening participants between 2002 and 2003 and subsequently underwent follow-up examinations up to 2013. NHIS-HEALS included information regarding participant eligibility (e.g., sex, age, and socioeconomic variables), health examination results (questionnaires on health-related behavioral variables and results of laboratory measurements), and a medical history (outpatient, inpatients, and pharmacy visits) for all visits to medical facilities of cohort sample. The NHIS-HEALS also includes the death registration information (e.g., causes and dates of deaths) from Statistics Korea. We cleaned the NHIS-HEALS data using the following three categories: (1) missing data- the study included missing values of variables that were used as confounding factors in the analysis models; (2) noisy data- there no cases of data repetition or data with key-in error; (3) inconsistent data- there were no inconsistencies in our data.

Among the 513,268 individuals who were included in the study between the years 2004 and 2007, we eliminated study participants with diagnosed type 1 diabetes from 2002 to 2007 (n = 6629); additionally, we excluded those without type 2 diabetes to include only those with type 2 diabetes as study subjects between 2004 and 2007 (n = 425,161). Type 2 diabetes was defined by the presence of any one of following criteria (n = 81,478): (1) fasting blood glucose level ≥ 126 mg/dL (7 mmol/L), (2) minimum 1 additional diagnosis of type 2 diabetes within 6 months following the initial date of the diagnosis under the International Classification of Diseases 10th Revision (ICD-10) codes (E11–E14), (3) prescription of anti-diabetes medication. We eliminated patients diagnosed with type 2 diabetes between 2002 and 2003 to recruit those with new-onset type 2 diabetes (n = 40,914). Furthermore, we excluded those who had a history of CVD before being diagnosed with sleep disturbance to minimize reverse causality (n = 4506) and the final study subjects were 36,058 patients with new-onset type 2 diabetes (Additional file 1: Fig. S1).

### Measurement

Outcome variable in this study was the presence of CVD and all-cause mortality. We used the ICD-10 codes as a primary diagnosis for CVD, which included CHD (I20–I25) and stroke (I60–I63). All-cause mortality was measured using the dates of mortality from the NHIS-HEALS database. The survival length was estimated in days, and all study participants underwent follow-up examination until the occurrence of either of the following three outcomes: incidence of CVD or mortality, withdrawal from the medical security system, or the end of 2013, whichever occurred first. We utilized sleep disturbance as independent variable and estimated the same using the G47 (sleep disorders) or F51 (sleep disorders not due to a substance or known physiological condition) under ICD-10 codes as a primary diagnosis [20].

Potential confounding factors in this study included sex, age, income level, area of residence, BP, BMI, fasting glucose, total cholesterol, physical activity, heavy alcohol consumption, smoking, family history of diabetes, and comorbidities. Sex, age, area of residence, and income level were measured using information from the year that included the index date. The index date was defined as initial diagnosis date of sleep disturbance following the diagnosis of type 2 diabetes. We used the initial date of diagnosing type 2 diabetes for those without sleep disturbance to define the same.

Age was classified to middle-aged adults (40–64 years) and older adults (≥ 65 years). Income level was classified as (1) low (< 40th percentile), (2) middle (41st–80th percentile), or 3) high (81st–100th percentile). Area of residence was classified as metropolitan (capital), urban (areas with a population of > 1 million), or rural (otherwise). Blood pressure (BP), body mass index (BMI), fasting glucose, total cholesterol, physical activity, heavy alcohol drinking, smoking, and family history of diabetes were identified through the data of a health screening closest to the index date. The recommendations of World Health Organization for Asian population were used to categorize study participants into five BMI groups: < 18.5 kg/m^2^ (underweight), 18.5–22.9 kg/m^2^ (normal), 23.0–24.9 kg/m^2^ (overweight), 25.0–29.9 kg/m^2^ (class I obese), or ≥ 30 kg/m^2^ (class II obese) [21]. Consuming ≥ 30 g/day of alcohol were defined as heavy alcohol consumption [22]. Physical activity was defined as exercising at least once a week. Smoking was categorized as non-smoking, ex-smoking, or currently smoking. Diastolic and systolic BP was measured after seating the individual for a minimum period of 5 min. Blood samples were obtained after overnight fasting to estimate serum glucose, and total cholesterol. Comorbidities were measured through the screening before the index date using medical records; they consisted of hypertension (ICD-10: I10–I15), dyslipidemia (ICD-10: E78), chronic kidney disease (ICD-10: N18), depression (ICD-10: F32–F33), chronic obstructive pulmonary disease (ICD-10: J43–J44 [except J43.0]), and cancer (ICD-10: C00–C99).

### Statistical analysis

Demographic and clinical characteristics in the study subjects were categorized according to sleep disturbance using a Pearson’s Chi square test and an independent *t* test. Data are indicated as number with percentage for categorical variables or as mean ± standard deviation for continuous variables. CVD and all-cause mortality were estimated for each study participant from the index dates between 2004 and 2007 to end of the study period on December 31, 2013. We measured the sum of person-years for follow-up and calculated the incidence of CVD and all-cause mortality per 10,000 person-years.

This study estimated adjusted hazard ratio (AHR) and 95% confidence interval (CI) with regard to the association between sleep disturbance and incidence of CVD and all-cause of mortality using Cox proportional hazards regression models. First, we examined the effect of sleep disturbance on the incidence of all CVD, coronary heart disease, stroke, and all-cause mortality among patients having new-onset type 2 diabetes. Second, we analyzed the relationship between sleep disturbance and risk of all CVD and all-cause mortality, and classified the resulting data by sex and age. Extraction and statistical analyses of the data were performed using the SAS 9.4 (SAS Institute Inc., Cary, NC). Proportional hazards assumptions were assessed statistically and satisfied for all models.

## Results

This study included 36,058 study subjects with newly diagnosed type 2 diabetes aged ≥ 40 years and examined 6897 events of all CVD (CHD, n = 4138; stroke, n = 2759) and 2890 events of all-cause mortality during a mean follow-up period of 7.0 ± 2.5 years. Table 1 presents the general characteristics of study participants according to sleep disturbance. The proportions of women (46.4% vs. 38.7%), older adults (43.4% vs. 24.6%), high income (32.6% vs. 29.9%), comorbidities (hypertension [56.7% vs. 40.9%], dyslipidemia [51.1% vs. 26.4%], depression [27.8% vs. 7.2%], chronic obstructive pulmonary disease [15.2% vs. 6.7%] and cancer [17.0% vs. 7.2%]) of individuals with sleep disturbance were significantly higher compared to those without sleep disturbance in patients with type 2 diabetes, respectively. However, those with sleep disturbances showed significantly lower levels of average systolic (128.9 mmHg vs. 131.1 mmHg) and diastolic (79.8 mmHg vs. 81.5 mmHg) BP, fasting glucose (114.2 mg/dL vs. 117.8 mg/dL), and the proportions of current smoking (16.6% vs. 22.3%), and physical activity (33.1% vs. 41.3%) of people with sleep disturbance were significantly lower compared to than those without sleep disturbance among patients with type 2 diabetes, respectively. Missing data for each confounding factors among study subjects has been presented in Additional file 1: Table S2. The results for compete case analysis were similar to our main findings (Additional file 1: Table S3).

**Table 1:** General characteristics of study subjects according to sleep disturbance

Variables	Total	Sleep disturbance				p-value
		Yes		No		
		N	%	N	%	
Total	36,058	870	2.4	35,188	97.6	
Women	14,030	404	46.4	13,626	38.7	< .001
Age (years)						< .001
40–64	27,009	492	56.6	26,517	75.4	
65≤	9049	378	43.4	8671	24.6	
BMI (kg/m^2^)						< .001
≤ 18.5	614	23	2.6	591	1.7	
18.5–23	8985	253	29.1	8732	24.8	
23–25	8678	184	21.1	8494	24.1	
25–30	12,658	246	28.3	12,412	35.3	
≥ 30	1511	33	3.8	1478	4.2	
BP (mmHg)						
Systolic	131.1 ± 17.5	128.9	17.3	131.1	17.5	< .001
Diastolic	81.4 ± 11.2	79.8	11.1	81.5	11.2	< .001
Fasting glucose (mg/dL)	117.8 ± 43.2	114.2	48.9	117.8	43.1	0.044
Total cholesterol (mg/dL)	203.1 ± 39.7	201.8	40.8	203.2	39.7	0.360
Family history of diabetes	2218	50	5.7	2168	6.2	0.897
Current smoking	8000	144	16.6	7856	22.3	0.002
Heavy alcohol drinking	2034	36	4.1	1998	5.7	0.113
Physical activity	14,825	288	33.1	14,537	41.3	< .001
Income level						0.020
Low	11,597	296	34.0	11,301	32.1	
Middle	13,641	290	33.3	13,351	37.9	
High	10,820	284	32.6	10,536	29.9	
Area of residence						0.341
Metropolitan	5547	129	14.8	5418	15.4	
Urban	9776	220	25.3	9556	27.2	
Rural	20,735	521	59.9	20,214	57.4	
Comorbidities						
Hypertension	14,877	493	56.7	14,384	40.9	< .001
Dyslipidemia	9726	445	51.1	9281	26.4	< .001
CKD	177	8	0.9	169	0.5	0.067
Depression	2776	242	27.8	2534	7.2	< .001
COPD	2488	132	15.2	2356	6.7	< .001
Cancer	2671	148	17.0	2523	7.2	< .001

BMI: body mass index; BP: blood pressure; CKD: chronic kidney disease; COPD: chronic obstructive pulmonary disease

Values are presented as mean ± SD or n (%)

Table 2 shows the AHR and 95% CI for incidence of CVD according to sleep disturbance in patients with new-onset type 2 diabetes. Following adjusting for sex, age, BMI, BP, fasting glucose, total cholesterol, family history of diabetes, smoking, heavy alcohol drinking, physical activity, income level, area of residence, and comorbidities, patients with sleep disturbance demonstrated a significantly increased risk of all CVD (AHR, 1.24; 95% CI, 1.06–1.46) than those without sleep disturbance among patients with type 2 diabetes. Regarding the CVD subtypes, the risk of CHD (AHR, 1.24; 95% CI, 1.00–1.53) increased significantly in patients with sleep disorders than those without, whereas study subjects with sleep disturbances was not significantly associated with the higher risk of stroke than those without sleep disturbances (AHR, 1.26; 95% CI, 0.98–1.63) in patients with type 2 diabetes. Patients with sleep disturbance were associated with increased risk of all-cause mortality significantly (AHR, 1.47; 95% CI, 1.15–1.87) than those without sleep disturbance among patients with type 2 diabetes.

**Table 2:** Association between sleep disturbance and incidence of CVD and all-cause mortality

Variables	N	Events	Person-years	The incidence of CVD or mortality per 10,000 person-years	HR^a^	95% CI		p-value
CVD events								
Sleep disturbance								
No	35,188	6694	247,987	269.9	1.00			
Yes	870	203	5075	400.0	1.24	1.06	1.46	0.009
CHD events								
Sleep disturbance								
No	35,188	4018	247,987	162.0	1.00			
Yes	870	120	5075	236.5	1.24	1.00	1.53	0.049
Stroke events								
Sleep disturbance								
No	35,188	2676	247,987	107.9	1.00			
Yes	870	83	5075	163.5	1.26	0.98	1.63	0.077
All-cause mortality								
Sleep disturbance								
No	35,188	2768	247,987	111.6	1.00			
Yes	870	122	5075	240.4	1.47	1.15	1.87	0.002

HR: hazard ratio; CI: confidence interval; CVD: cardiovascular disease; CHD: coronary heart disease

^a^HRs were estimated after adjusting for sex, age, BMI, BP, fasting glucose, total cholesterol, family history of diabetes, smoking, heavy alcohol drinking, physical activity, income level, area of residence, and comorbidities

**Fig. 1:**
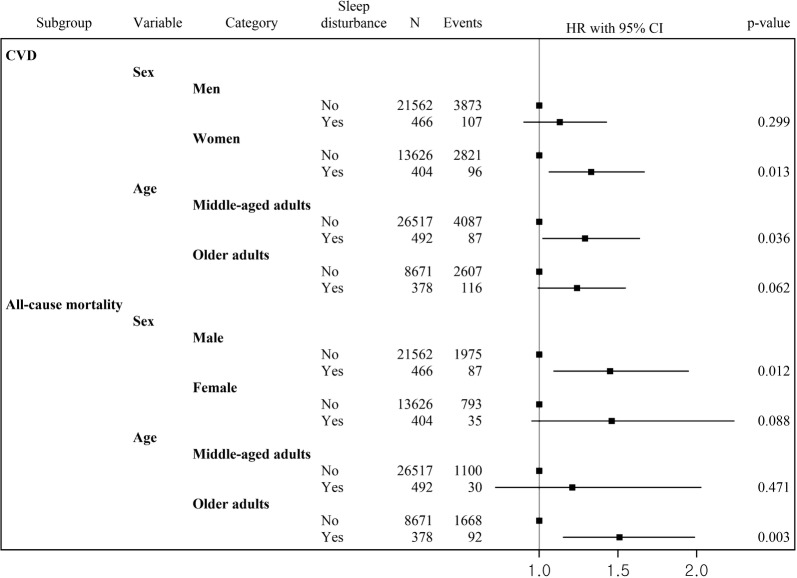
Association between sleep disturbance and incidence of CVD and all-cause mortality according to sex and age

Figure 1 shows the results for association between sleep disturbance and incidence of CVD and all-cause mortality according to sex and age. In patients with type 2 diabetes, women (AHR, 1.33; 95% CI, 1.06–1.67) and middle-aged adults (AHR, 1.29; 95% CI, 1.02–1.64) with sleep disturbance showed a significantly higher risk of CVD than those without sleep disturbance; however, the risk of all-cause mortality increased significantly in men (AHR, 1.45; 95% CI, 1.09–1.95) and older adults (AHR, 1.51; 95% CI, 1.15–1.99) with sleep disorders than those without sleep disturbance.

## Discussion

Sleep disturbance was associated with an increased risk for all CVD and CHD events, along with all-cause mortality in patients with new-onset type 2 diabetes. In patients with type 2 diabetes, women and middle-aged adults with sleep disturbance showed a significantly higher risk of CVD than those without, whereas the risk of all-cause mortality significantly increased in men and older adults with sleep disturbance than those without sleep disturbance.

Despite the presence of a growing amount of evidence regarding the relationship between sleep disturbances and CVD, most of these studies have primarily focused on a population of individuals with a normal glucose tolerance. Additionally, most prior studies have utilized sleep duration as the only measure of sleep. However, a more recent evidence demonstrated the importance of studying parameters of sleep other than sleep duration [23, 24]. Our study differs from previous studies by investigating the correlation between sleep disturbance and CVD risk in type 2 diabetes.

Our findings indicated that sleep disturbance was associated with a higher risk of CVD in patients with type 2 diabetes, which was consistent with the outcomes of previous studies [18]. Sleep disturbance could activate the autonomic nervous system and promote the secretion of catecholamine, which is a well-known risk factor for CVD [25]. Furthermore, sleep disturbance may also activate pro-inflammatory transcription factors such as nuclear factor kappa B (NF-κB), followed by elevating the expression of adhesion molecules (such as ICAM-1, VCAM-1 and selectins) and pro-inflammatory mediators (such as TNF-α, IL-6 and IL-8). Subsequently, it may activate numerous endothelial cells and inflammatory cells, which may result in endothelial dysfunction. Endothelial dysfunction considerably influences CVD incidence [26].

Obstructive sleep apnea is frequently observed in cases of metabolic syndrome [27] and it is one of the mechanisms that increase the risk of new-onset atrial fibrillation in obese individuals [28]. Divergence from the recommended 7 to 8 h of sleep is associated with a higher risk of mortality and cardiovascular events. Longer duration of sleep may be more associated with adverse outcomes compared with shorter sleep durations [11], concurrently, individual and coexisting symptoms of insomnia are independent risk factors for CVD incidence, particularly in young adults or adults without hypertension [29].

Sleep disturbance and all-cause mortality were significantly associated with each other in general population. Previously, a study demonstrated that individuals who had difficulty in falling asleep had 28% greater risk of all-cause mortality [30]. A meta-analysis study revealed the correlation between obstructive sleep apnea and an increased risk of all-cause mortality [31]. Our findings further validated those reported previously by extending the findings to patients with type 2 diabetes.

Our findings showed that sleep disturbance was associated with higher risk of coronary heart disease and not related to stroke. Concerning stroke, our findings are in the context of a literature that has shown conflicting results—although several studies indicated a significant association of a short sleep duration [32] and sleep apnea [33] with stroke, a study indicated the presence of a non-significant association between sleep disturbance and stroke [34]. Further research is needed to explore the association between sleep disturbance and stroke in type 2 diabetes.

Our findings suggested that sleep disturbance was associated with higher risk of all-cause mortality in men and older adults with type 2 diabetes, which is consistent with previous study findings [35, 36]. However, this study revealed that sleep disturbance was associated with higher risk of all CVD in men and older adults with type 2 diabetes, which is not in line with previous studies. Sabanayagam and Shankar implied that excessively short or long sleep durations were associated with a higher risk of CVD in men, women, middle-aged and older adults, respectively [32]. Amagai et al. suggested that men who slept for fewer than 6 h a day were at an increased risk of CVD [37]. Our results implied that the association between sleep problems and all CVD according to age and sex among patients with type 2 diabetes was different from that of the general population and further research would be needed to examine these differences.

Our study has several potential limitations. First, we could not evaluate the association between subtypes of sleep disturbance and CVD and mortality because of limited data (Additional file 1: Table S1). Additionally, we could not assess more sleep-related factors including sleep duration, sleep depth, sleep timing, and degree of sleep disturbance since we used secondary data. Future research is warranted to explore CVD and mortality risk according to the subtypes and specific characteristics of sleep disturbance using adequate data. Second, although we excluded subjects diagnosed with CVD before being diagnosed with sleep disturbance, there may have been cases of reverse causality. Third, diagnosis of type 2 diabetes, sleep disturbance, comorbidities, and cardiovascular disease was identified according to ICD-10 codes of the NHIS claims database without reviewing the detailed clinical charts. The misclassification based on ICD-10 codes may have potentially affected our findings. Fourth, although the results of a compete case analysis were similar to our main findings, the missing data may potentially influence our findings. Fifth, biomarkers such as catecholamine, nuclear factor kappa B were not considered in this study due to the lack of pertinent data. Further studies on the association between sleep disturbance and CVD in patients with type 2 diabetes need to incorporate these biomarkers. Finally, we only included participants from the Korean population. Therefore, further investigation is needed to verify that our findings can be applied to individuals of other ethnicities.

## Conclusions

This is possibly the first study to explore the association between sleep disturbance and risk of CVD and all-cause mortality in patients with new-onset type 2 diabetes. This study verified the significant association between sleep disturbance and an increased risk of CVD and mortality among patients with new-onset type 2 diabetes. Our results have implied the need for a more scrupulous investigation and appropriate management of sleep disturbance in the patients with type 2 diabetes to partly prevent the incidence of CVD events and premature mortality.

## Supplementary information

**Additional file 1: Figure S1.** Flow chart of the study participants. **Table S1.** The number of incidence of CVD and all-cause mortality according to subtypes of sleep disturbance. **Table S2.** The number of missing values for potential confounding factors in this study. **Table S3.** Association between sleep disturbance and incidence of CVD and all-cause mortality in subjects with complete information for confounding factors.

## Data Availability

Data may be obtained from a third party and are not publicly available

## Abbreviations

CVD: Cardiovascular disease
NHIS-HEALS: National Health Insurance Service–Health Screening Cohort
ICD-10: The International Classification of Diseases 10th Revision
BP: Blood pressure
BMI: Body mass index
AHR: Adjusted hazard ratio
CI: Confidence interval
