# Supplementary material for: Association of sleep disturbance with risk of cardiovascular disease and all-cause mortality in patients with new-onset type 2 diabetes: data from the Korean NHIS-HEALS
Source: Cardiovasc Diabetol. 2020 May 13;19:61. doi: 10.1186/s12933-020-01032-5 (PMC7222449; doi:10.1186/s12933-020-01032-5)
Supplement: Supplementary file 1 — Additional file 1: Figure S1. Flow chart of the study participants. Table S1. The number of incidence of CVD and all-cause mortality according to subtypes of sleep disturbance. Table S2. The number of missing values for potential confounding factors in this study. Table S3. Association between sleep disturbance and incidence of CVD and all-cause mortality in subjects with complete information for confounding factors. [file 12933_2020_1032_MOESM1_ESM.docx]

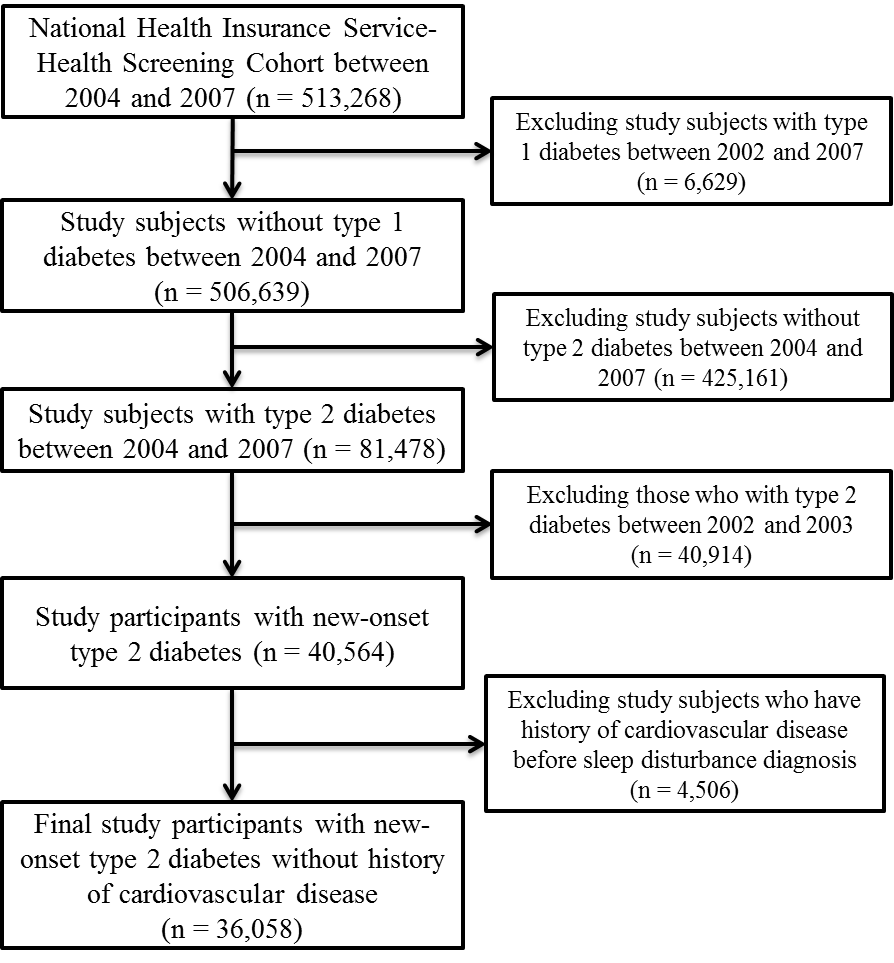


Figure S1. Flow chart of the study participants

| Table S1. The number of incidence of CVD and all-cause mortality according to subtypes of sleep disturbance | | | | | |
| --- | --- | --- | --- | --- | --- |
| ICD-10 codes | Definition | CVD | | All-cause mortality | |
|  |  | Yes | No | Yes | No |
| F510 | Insomnia not due to a substance or known physiological condition | 78 | 234 | 43 | 269 |
| F511 | Hypersomnia not due to a substance or known physiological condition | 0 | 2 | 0 | 2 |
| F512 | Nonorganic disorder of the sleep | 0 | 2 | 0 | 2 |
| F513 | Sleepwalking | 0 | 0 | 0 | 0 |
| F514 | Sleep terrors | 0 | 0 | 0 | 0 |
| F515 | Nightmare disorder | 0 | 0 | 0 | 0 |
| F518 | Other sleep disorders not due to a substance or known physiological condition | 1 | 11 | 0 | 12 |
| F519 | Sleep disorder not due to a substance or known physiological condition, unspecified | 11 | 34 | 7 | 38 |
| G470 | Insomnia | 78 | 263 | 51 | 290 |
| G471 | Hypersomnia | 2 | 0 | 0 | 2 |
| G472 | Circadian rhythm sleep disorders | 0 | 0 | 0 | 0 |
| G473 | Sleep apnea | 11 | 40 | 2 | 49 |
| G474 | Narcolepsy and cataplexy | 0 | 1 | 0 | 1 |
| G475 | Parasomnia | 0 | 0 | 0 | 0 |
| G476 | Sleep related movement disorders | 0 | 0 | 0 | 0 |
| G478 | Other sleep disorders | 1 | 13 | 3 | 11 |
| G479 | Sleep disorder, unspecified | 21 | 67 | 16 | 72 |
| Total | | 203 | 667 | 122 | 748 |

| Table S2. The number of missing values for potential confounding factors in this study | | | | | |
| --- | --- | --- | --- | --- | --- |
| Variables | | The number of missing values | | | |
|  |  | With sleep disturbance | | Without sleep disturbance | |
|  |  | N | % | N | % |
| Total | | 870 | 100.0 | 35,188 | 100.0 |
| Sex | | 0 | 0.0 | 0 | 0.0 |
| Age (years) | | 0 | 0.0 | 0 | 0.0 |
| BMI (kg/m^2^) | | 131 | 15.1 | 3,481 | 9.9 |
| SBP (mmHg) | | 131 | 15.1 | 3,477 | 9.9 |
| DBP (mmHg) | | 131 | 15.1 | 3,478 | 9.9 |
| Fasting glucose (mg/dL) | | 132 | 15.2 | 3,484 | 9.9 |
| Total cholesterol (mg/dL) | | 132 | 15.2 | 3,513 | 10.0 |
| Family history of diabetes | | 210 | 24.1 | 6,055 | 17.2 |
| Current smoking | | 167 | 19.2 | 4,969 | 14.1 |
| Heavy alcohol drinking | | 144 | 16.6 | 4,036 | 11.5 |
| Physical activity | | 151 | 17.4 | 4,228 | 12.0 |
| Income level | | 0 | 0.0 | 0 | 0.0 |
| Area of residence | | 0 | 0.0 | 0 | 0.0 |
| Comorbidities | |  |  |  |  |
|  | Hypertension | 0 | 0.0 | 0 | 0.0 |
|  | Dyslipidemia | 0 | 0.0 | 0 | 0.0 |
|  | CKD | 0 | 0.0 | 0 | 0.0 |
|  | Cancer | 0 | 0.0 | 0 | 0.0 |
| Note. BMI: Body Mass Index; SBP: Systolic Blood Pressure; DBP: Diastolic Blood Pressure; CKD: Chronic Kidney Disease | | | | | |

| Table S3. Association between sleep disturbance and incidence of CVD and all-cause mortality in subjects with complete information for confounding factors | | | | | | | | |
| --- | --- | --- | --- | --- | --- | --- | --- | --- |
| Variables | | | N | Events | HR^a^ | 95% CI | | p-value |
|  |  |  |  |  |  |  |  |  |
| CVD events |  |  |  |  |  |  |  |  |
|  | Sleep disturbance | |  |  |  |  |  |  |
|  |  | No | 27,420 | 5,105 | 1.00 |  |  |  |
|  |  | Yes | 622 | 152 | 1.26 | 1.07 | 1.48 | 0.006 |
| CHD events |  |  |  |  |  |  |  |  |
|  | Sleep disturbance | |  |  |  |  |  |  |
|  |  | No | 27,420 | 3,120 | 1.00 |  |  |  |
|  |  | Yes | 622 | 90 | 1.26 | 1.02 | 1.50 | 0.035 |
| Stroke events |  |  |  |  |  |  |  |  |
|  | Sleep disturbance | |  |  |  |  |  |  |
|  |  | No | 27,420 | 1,985 | 1.00 |  |  |  |
|  |  | Yes | 622 | 62 | 1.26 | 0.98 | 1.63 | 0.073 |
| All-cause mortality | |  |  |  |  |  |  |  |
|  | Sleep disturbance | |  |  |  |  |  |  |
|  |  | No | 27,420 | 1,857 | 1.00 |  |  |  |
|  |  | Yes | 622 | 70 | 1.53 | 1.20 | 1.94 | <.001 |
| Note. HR, hazard ratio; CI, confidence interval; CVD, cardiovascular disease; CHD, coronary heart disease | | | | | | | | |
| ^a^HRs were estimated after adjusting for sex, age, BMI, BP, fasting glucose, total cholesterol, family history of diabetes, smoking, heavy alcohol drinking, physical activity, income level, area of residence, and comorbidities | | | | | | | | |
